# Preanesthetic Evaluation and Assessment of Children with Down's Syndrome

**DOI:** 10.1100/tsw.2007.64

**Published:** 2007-02-19

**Authors:** Letterio B. Santamaria, Carmelo Di Paola, Federica Mafrica, Vincenzo Fodale

**Affiliations:** ^1^ Department of Neurosciences, Psychiatric and Anesthesiological Sciences, University of Messina, Via C. Valeria, Messina, Italy; ^2^ Department of Anesthesia, I.R.C.C.S., Oasi Maria S.S. di Troina (EN), Italy

**Keywords:** Preanesthetic evaluation, preoperative testing, evaluating anesthetic risk, Down’s syndrome, mental retardation

## Abstract

During preoperative evaluation for anesthesia in the Down patient, it is important to focus attention on the functional conditions of the patient and systems that frequently show anomalies. One of the challenges of evaluating pre-operative conditions and potential risks in the Down patient is the lack of a gold-standard evaluation score; cervical spine abnormalities, reduced dimensions and malformations of the airways, neurological changes, respiratory and cardiac disease, as well as endocrinological and metabolic alterations. We suggest, as a possible method of evaluation for patients with mental retardation and possible malformations, a new scale which takes the functional and mental conditions into account: the Sensorial, Psychological, Anatomical, Biological, Operational and Surgical (SPABOS) Compliance Score.

## INTRODUCTION

Perioperative responsibility is divided between anesthesiologists and surgeons, with the anesthesiologist responsible for preoperative risk evaluation, perioperative management, and maintenance of vital organ functions. The aims are patient safety, efficient preoperative evaluation and subsequent patient optimization to reduce the burden of complications on the health care system[1]. Therefore, the ultimate goal of preanaesthetic evaluation and preparation of the patient is the reduction of morbidity and mortality during and after anesthesia and surgery[2].

Preanaesthetic evaluation is an essential element of the anesthetic management of a patient. The type of practice, academic or private, should have no bearing on the quality of the evaluation. The practice environment may play a significant role in the type of patient and procedure but should not reduce the quality of care for any particular patient[3].

Understanding and stratifying the perioperative risks allows the anesthesiologist to develop a systematic, focused approach to these patients at the time of the initial contact and immediately before induction, which can be used to guide anesthetic management[4]. In addition, assessments made during the preanesthetic evaluation may be used to educate the patient, organize resources for perioperative care, formulate plans for intraoperative care, postoperative recovery, and perioperative pain management[5]. Therefore, a complete preanaesthetic evaluation is the most important function an anesthesiologist can perform to prevent anesthetic morbidity and mortality and ensure a smooth course of anesthetic administration[6] .

The basis of any preoperative medical evaluation is a thorough history and physical examination of the patient. The history should include a complete review of systems (especially cardiovascular and pulmonary), medication, allergies, surgical and anesthetic history, and functional status[7]. A careful medical history, evaluation and physical examination in apparently healthy individuals can avoid extensive laboratory screening tests; however, in the case of pathological findings, specific diagnostic procedures should ensue[8]. Where possible, preoperative therapeutic interventions must be considered to reduce the risk associated with concomitant diseases[9]. By using the preanesthetic assessment as an opportunity to provide comprehensive teaching about the entire perianesthetic process, the patient and family are prepared for the best possible outcome[10].

## Preanesthetic Evaluation in Down’s Syndrome

Down’s syndrome is the most common form of intellectual disability in the Western World. The syndrome is characterized by congenital malformations, especially of the heart and gastrointestinal tract, which can result in high mortality rates in the affected population. Preoperative evaluation for anesthesia, in patients with Down’s syndrome, must include an adequate evaluation of the airways, neurological status, pulmonary and cardiac function, as well as endocrinological dysfunctions with an emphasis on the thyroid gland, and pancreas. Many improvements have been made in the medical treatment of this syndrome during the past few decades, and the survival of individuals with Down’s syndrome has increased in the industrial world[11].

Also, improvements in the quality of life of these individuals result from improvements in identification and treatment of psychiatric disorders (such as depression, disruptive behavior disorders, and autism), and early educational interventions with support in typical educational settings[12].

In addition, cervical spine radiography must be done to rule out possible atlanto-axial instability (AAI).

## Role of Cervical Spine Radiography

Cranio-cervical junction abnormalities are a frequent finding in persons with Down’s syndrome[13]. Atlanto-axial instability has been observed in a significantly greater number of children with Down syndrome[14]. Twenty-eight subjects with Down’s syndrome, 9-29 years old, were examined for instability of the cervical spine at the atlanto-axial and atlanto-occipital joints. Clinical goniometric measurement demonstrated specific and significant increases in the extension movement of Down patients who presented with one of these alterations[15].

While neurological symptoms occur less frequently, fatal atlanto-axial instability with spinal cord compression does occur in Down’s syndrome after minor trauma of the cervical spine[16]. Widening of the anterior atlanto-odontoid distance (AAOD), and atlanto-occipital instability occur in up to 21% and 63% of Down patients, respectively, but neurological complaints are uncommon and rarely severe enough to contribute to the patient's demise[17]. In a study on atlanto-occipital mobility, performed in 57 patients with Down’s syndrome, it has been observed that this alteration is asymptomatic in the majority of the subjects with Down’s syndrome, but the possibility of neurologic complications should be considered[18].

Twenty-seven Down patients have been examined to establish the atlanto-occipital instability frequency. In two of them there was an atlanto-axial dislocation, but without any corresponding neurological symptoms. However, examination of the X-rays revealed many cases with marked degenerative changes, particularly in the upper part of the cervical spine[19].

A review of the radiographs of 34 individuals with Down's syndrome, between 5 and 21 years of age, demonstrated subluxation of atlantoaxial instability (C1-C2) greater than 5 mm in three of the 34 individuals (9%). The authors suggest that cervical spine radiography be carried out on all Down children older than 5 years of age, and should also be considered in all Down adults, particularly those 30 years of age and older[20].

Moreover, during a study on X-rays of the cervical spine of 220 Down suffers of all ages, the authors noted that there were degenerative changes in the cervical spine at an early age in Down patients, and there is both an increased incidence and severity of degenerative change at higher levels with increasing age[21]. So, it may be that atlanto-occipital instability presents degenerative changes with increasing age, and for this reason it is advisable to repeat the radiograph studies if, later on, the patient returns to the operating room.

An analysis of lateral radiographs of cervical spine in 31 children with Down's syndrome revealed 8 cases (nearly 25%) of substantial anomalies (anterior subluxation of the apical vertebrae in 3 cases and C1 laminar hypoplasia in 5 cases)[22]. As part of a study to determine whether patients with Down's syndrome should be screened for atlantoaxial instability before they participate in sport, a group of 279 children, aged 6 to 17 years was investigated radiologically. Sex and age together explained, at most, 9% of the variation in atlantoaxial distance. The maximum distance found was 6.5 mm. The difference between the means of first and second measurements by the same (test-retest) and by another (inter-) observer was greater for those taken in the neutral position than in flexion[22]. Anteroposterior occipitoatlantal hypermobility was observed in patients with Down's syndrome only when atlantoaxial instability was present [23,24]. Therefore, all patients with Down's syndrome should have a preoperative neurologic assessment screening by the operating surgeon and/or a cervical roentgenogram in the lateral, extension, and flexion positions. Any abnormality should be investigated before surgery[25].

In addition, some authors have recommended cervical spine radiographs prior to elective surgery in all children with trisomy-21. In a study regarding the perioperative evaluation and management of potential cervical spine instability in patients with trisomy-21, it has been demonstrated that the majority of respondents base their preoperative evaluation of the cervical spine on the signs and symptoms of the patient[26].

Clinical signs and symptoms that might predict atlantoaxial subluxation were studied prospectively in 135 out of 180 children with Down's syndrome, aged 6-14 years, from the Hester Adrian Research Centre cohort. The radiographs of the cervical spine are unreliable for identifying atlantoaxial subluxation in children with Down's syndrome, and no reliable clinical predictor could be identified[27].

## Other Alterations Regarding Joints

Down subjects present anomalies that involve other joints and this may interest the anesthesiologist, particularly regarding the position of the patient on the operating table.

The biomechanical characteristics of the ankle during gait of 17 participants with Down's syndrome, ages 8 to 36 years, were investigated. Correlation between kinetic and temporal spatial parameters was markedly reduced or weak. These results point to a hypofunctioning of the ankle, probably due to hypotonia and ligament laxity[28].

Cinematographic analysis techniques were used to evaluate the walking patterns of 10 5-year-old Down's syndrome (DS) children (trisomy 21). The DS children adopted a more flexed posture of the hip and knee joints, and increased fluctuation of ankle movement during the walking cycle. The data suggest a wide continuum of locomotor development which may be a function of the degree of impairment of associated neuromuscular mechanisms[29].

## Why is The Preoperative Management of The Airways in Down's Syndrome Important?

The number of patients with both tracheal stenosis with hourglass trachea, and midtracheal absence of the tracheal pars membranacea suggests that the association of this pattern of congenital tracheal stenosis, together with Down's syndrome is, although infrequent, significant[30].

The inner diameter of the tracheal air column was measured at 2 cm above the aortic arch in 14 adult patients with Down's syndrome, and the results were compared with previously established norms. Tracheal diameters in adult patients with Down's syndrome are reduced and the narrowing cannot be ascribed to associated congenital heart disease or to body habitus[31]. This is because of an overall decrease in the diameter of the tracheal lumens. Initial intubation of a child with Down's syndrome should be performed with an endotracheal tube at least two sizes smaller than would be used in a child of the same age without Down's syndrome, to avoid potential airway trauma[32]. In addition, residual symptoms of airway obstruction in children with Down's syndrome are common after surgery. Therefore, a comprehensive and individualized approach is important in the management of the patient with this syndrome[33].

The clinical presentation, frequency, and type of airway anomalies in a population of patients with DS has been characterized. A retrospective evaluation of flexible bronchoscopies performed in 24 DS patients due to significant respiratory morbidity was compared to the findings in 324 non-DS patients. The most important endoscopic findings were: laryngomalacia (12/24), tracheomalacia (8/24), tracheal bronchus (5/24), and bronchomalacia (5/24). Only six patients had a normal examination. Patients with DS and respiratory symptoms had a high incidence of airway anomalies compared to non-DS patients. The clinician should have a high index of suspicion for airway anomalies in DS patients with respiratory symptoms[34].

In addition, Down patients with airway obstructions may be completely asymptomatic in the early months of life[35].

## Cognitive and Cerebral Problems Related to Down's Syndrome

Neurological abnormalities concern neuroanatomy and neurotransmission. In addition to severe learning difficulties, Down's syndrome individuals have craniofacial abnormalities, clinical defects of the heart, gut and immune system, as well as Alzheimer's disease[36]. Abnormalities in the structure of nerve cells have been observed in the neuroaxis, cerebellum and central structures. The brain of individuals over the age of 40 exhibits the characteristic neuropathologic and neurochemical stigmata of Alzheimer's disease. With regard to alterations of the neurotransmission system, the cholinergic and noradrenergic systems appear to be particularly vulnerable[37].

Moreover, it has been observed that individuals with Down's syndrome have decreased perception of pain, which could be related to raised levels of opioid peptides in the frontal cortex of these people. Increased concentration of the analgesic opioids leu-enkephalin and dynorphin A, in an area of the brain involved with integrating physiological responses to environmental stimuli, could explain the increased pain threshold in Down's syndrome patients[38,39].

Also, a subnormal peripheral somatosensory function might include the transmission of painful stimuli that result, in addition to other dysfunctions of this syndrome, from the acquisition of trisomy 21[40].

These alterations may contribute to neurodegeneration and dementia development in this syndrome, and may have consequences in conduction of anesthesia and administration of anesthetic drugs. In addition, an altered pain tolerance, and the incapacity to give a qualitative and quantitative description, have emerged in a powerful way, which causes difficulty in measuring pain in Down patients.

The syndrome is the most common single cause of mental retardation[41] which is characterized by developmental delays, language and memory deficits and other cognitive abnormalities. Subjects with Down's syndrome, as a group, are less accurate in location memory than college students, but many individuals perform just as accurately. Location memory is the memory for spatial location, and has frequently been investigated in mentally retarded populations[43]. A 3-month follow-up on subjects with Down's syndrome revealed greater consistency in location memory than in recall[42].

The diagnosis of dementia, which is difficult to establish, requires a process which is consistent over time, underlining the influence of the context and the human environment on the aging of the Down's syndrome subject[44]. It may be difficult to distinguish between cognitive deterioration and various degrees of pre-existing intellectual disability.

The Prudhoe Cognitive Function Test (PCFT) provides a reliable quantitative measure of cognitive function in subjects with Down's syndrome, and could be a useful adjunct to the diagnosis of dementia in prospective studies. However, the almost uniformly low scores obtained by those with high levels of intellectual disabilities suggest that its power to detect cognitive decline will be limited to those who are less disabled, while the Adaptive Behavior Scale may be more useful than the PCFT in detecting deterioration in people with profound intellectual disabilities[45].

Although epilepsy is more common in persons with trisomy 21 than in the general population, the mechanisms by which seizures are generated in this population have received little attention. It is likely that this increased seizure susceptibility is due to a combination of medical risk factors and inherent neurologic abnormalities characteristic of Down's syndrome[46].

With regard to the use of anesthetic drugs in Down patients with epilepsy, thiopental is usually administered instead of propofol, while for dementia in Down patients, the same technique is used as with Alzheimer's disease.

## Respiratory System Diseases in Down's Syndrome

Chronic sinusitis, otitis with secretions, and upper respiratory tract infections are commonly found in patients with Down's syndrome. These diseases are generally felt to be secondary to depressed immune function, altered craniofacial dimensions and recently, to primary cilia ultrastructure abnormalities[47]. The nature of the mucociliary defect in Down's syndrome has been attributed to recurrent respiratory tract infections causing changes in mucus properties, such as in rheological parameters, and not only to a primitive defect of cilia[48].

Down's syndrome is considered a risk factor for pulmonary vascular disease (PVD). Children with Down's syndrome frequently have obstructive sleep apnea syndrome (OSAS), with obstructive sleep apnea, hypoxemia, and hypoventilation. OSAS may contribute to unexplained pulmonary hypertension seen in children with Down's syndrome[49]. Chronic airway obstruction may lead to pulmonary artery hypertension (PAH) that is more likely to develop in premature infants or children with Down's syndrome and cardiac anomalies. Surgery or supplemental oxygen will usually improve PAH, but fixed and irreversible PAH has been known to develop in patients with severe airway disease[50].

With regard to cardiorespiratory capacity, Down patients have even lower levels of peak oxygen uptake (peak $VO_2$) than subjects without the syndrome, a finding that is possibly mitigated by the lower peak heart rates of individuals with Down's syndrome[51]. Alveolar hypoplasia is characteristic of the syndrome. Distension of peripheral air spaces or interstitial emphysema, due to artificial inflation of the lungs during surgery, has been reported. The severity of lesions is significantly correlated to the duration of artificial respiration and severity of respiratory failure. Hypoplastic lung tissue in patients with Down's syndrome appears to be more susceptible to mechanical stress, and this is likely to be the cause of postoperative respiratory failure[52].

Preoperative cardiac ultrasonography is important to determine the presence of right-sided heart failure, which may be an indication for cardiac catheterization to determine pulmonary venous pressures.

## Cardiovascular System in Down's Syndrome

Congenital cardiac malformations are frequently associated with non-cardiac malformations and chromosomal anomalies[53]. The frequency of congenital heart disease in children with Down's syndrome is high, ranging between 40 - 50% of cases. Nevertheless, patients with trisomy 21, despite several congenital heart defects, appear to be "protected" from other defects (situs inversus and situs ambiguus, ventricular inversion, transposition of the great arteries)[54]. The most frequent cardiac malformations are interauricular septal defect (IASD), atrioventricular septal defect (AVSD) interventricular septal defect (IVSD) and patent ductus arteriousus (PDA) (90%)[55]. With regard to acute ischemic disease of myocardium, myocardial infarction is rare in patients with Down's syndrome[56]. The lipid and lipoprotein levels observed in the population cannot explain the decreased prevalence of coronary artery disease. In some studies, triglyceride levels were significantly increased, and serum HDL cholesterol, apo AI and HDL cholesterol: total cholesterol ratio were significantly decreased[57].

### Endocrinological, Metabolic, and Kidney Dysfunction in Down’s Syndrome

Patients with Down's syndrome with multiple autoimmune phenomena are common. They suffer from celiac-like enteropathy and hemolytic anemia, and display cellular immunity directed against peripheral nerve antigen and basic myeloprotein, and serum autoantibodies for many other tissue antigens[58]. These autoimmune disorders become increasingly frequent as the patients grow older, and the onset of one autoimmune disease often predisposes the development of others. Autoimmune thyroiditis is the most frequent disorder and appears to affect 39% of adult patients with Down’s syndrome[59].

Clinical and subclinical thyroid dysfunction is frequent in patients with Down’s syndrome and risk increases with age. Hypothyroidism is the most frequent dysfunction but hyperthyroidism is also associated with Down’s syndrome[60]. The general reduction in thyroid function in Down individuals may be due to impaired development of the thyroid gland. However, clear chemical hypothyroidism may occur only when thyroiditis is superimposed on a pre-existing diminished thyroid reserve[61]. Therefore, surveillance with yearly TSH measurements should be carried out in these patients, since signs and symptoms of thyroid disease are rarely detected. An association between autoimmune thyroid disease and diabetes mellitus has been well recognized. It is likely that the generalized autoimmune disorder is the underlying cause[62]. These facts, together with abnormalities which occur in Down’s syndrome glycolysis, make phosphofructokinase (PFKL) overexpression a candidate for causing some aspects of the Down’s syndrome phenotype[63].

Kidney disease has not been considered a frequent complication in Down’s syndrome patients, but a variety of urological abnormalities and glomerulopathies have been reported in this population, and some Down patients develop chronic renal failure (CRF)[64]. Understanding the underlying causes of renal failure could potentially lead to prevention of progressive renal dysfunction in this population. Monitoring of Down patients for renal, and especially glomerular disease, should be carried out regularly as patients age into the second and third decades[65].

## THE SENSORIAL, PSYCHIC, ANATOMICAL, BIOLOGICAL, OPERATION AND SURGICAL

### (SPABOS) Compliance Score

Based on the above-mentioned data, patients with Down’s syndrome may experience alterations and disease in a combined manner not found in patients without the syndrome. Consequently, the anesthesiologist who works with these patients could experience difficulties in the evaluation and classification of the potential risk related to the surgical operation and anesthesia. The common, available methods used for pre-operative evaluation (i.e. American Society of Anesthesiologist or ASA Score) are unable to present and describe the real status and alterations often presented by these patients in term of

**TABLE 1**
**Sensorial, Psychic, Anatomical, Biological, Operation and Surgical (SPABOS) Compliance Score is of value for subjects with diseases involving anatomical alteration and mental retardation.**

| | | | |
|---|---|---|---|
| Sensorial Status: | Value the sensorial status. | Intact | (0) |
| | | Drowsy | (1) |
| | | Incomplete sensorium | (2) |
| | | State of excitement | (3) |
| Psychic Conditions: | Consider the collaboration that the patient offers. | Collaborating | (0) |
| | | Low collaboration | (1) |
| | | Non-collaborating | (2) |
| | | Opposing | (3) |
| Anatomical and functional alterations: | Value the presence of anatomical and skeleton alterations. | Absent | (0) |
| | | Slight | (1) |
| | | Moderate | (2) |
| | | Severe | (3) |
| Biological Compliance: | Diseases with general involvement. | Absent | (0) |
| | | Present but compensated | (1) |
| | | Low compensation | (2) |
| | | Non-compensated | (3) |
| Operation Duration: | Presumed duration of operation. | Few minutes | (0) |
| | | Less than two hours | (1) |
| | | Up to four hours | (2) |
| | | Operations of long duration | (3) |
| Surgical Damage: | Presumed or sure possibility that the operation may cause damage to the respiratory, cardiovascular, hepatic or metabolic functions. | No damage | (0) |
| | | Slight damage | (1) |
| | | Serious damage | (2) |
| | | Damage to many functions | (3) |

| | | |
|---|---|---|
| 0 | No risk added | TOTAL SCORE |
| 1-4 | Low risk | |
| 5-7 | Moderate risk | S...+ P...+ A...+ B...+ O...+ S... = |
| 8-10 | Average risk | |
| 10-18 | High risk | |
| 19-24 | Patient with very elevated risk | |

NOTE: We use low, moderate or severe, to describe the gravity of the alteration of anatomy or function of considered system or apparatus on the basis of anamnesis and laboratories data, and exams of instrumental diagnosis, while this Score is based on quantity assessment.

psychic, sensorial, and biological conditions. In addition, the scores commonly used in the preoperative evaluation do not consider the duration and type of surgery.

Due to congenital anomalies of various systems and apparatus, Down patients present an elevated risk of intraoperative hypoxemia, intra- and postoperative death, and postoperative aggravation of their clinical condition. There is difficulty in applying, the best anesthetic technique because of difficulties in proper endotracheal tube positioning, maxillo-facial anomalies, and first airway and atlanto-occipital instability. To evaluate these risk, an "*ad hoc*" new pre-operative evaluation score has been introduced in the clinical practice: the SPABOS Compliance Score, that considers six different parameters (Table 1) sensorial status, psychic conditions, anatomic and functional alterations, biological compliance, operation duration and surgical damage. The new score is a modification and evolution of a previous preoperative classification proposed by Dr. Di Paola and Colleagues, and in use for several years in the pre-operative management of subjects with Down's syndrome at the Clinical Scientific Research Institute *I.R.C.C.S.* Oasi Maria S.S. of Troina (Enna, Italy), a surgical and medical centre for patients with Down's syndrome and diseases with mental retardation. This score is also of value for subjects with diseases involving anatomical alteration and mental retardation.

## CONCLUSIONS

Pre-operative examination for patients with Down's syndrome should aim at a careful evaluation of organs and of systems which present malformations characteristic of this syndrome. The anesthetist has a very important role, since he must establish a relationship between patient status and perioperative risk. Therefore, the Psycho-Sensorial Biological and Surgical Compliance Score is a useful, practical method of evaluation, helping the physician evaluate the complexity of these patients' more easily.
